# From a Well-Defined Organozinc Precursor to Diverse Luminescent Coordination Polymers Based on Zn(II)-Quinolinate Building Units Interconnected by Mixed Ligand Systems

**DOI:** 10.3390/molecules26237402

**Published:** 2021-12-06

**Authors:** Katarzyna Sołtys-Brzostek, Kamil Sokołowski, Iwona Justyniak, Michał K. Leszczyński, Natalia Olejnik-Fehér, Janusz Lewiński

**Affiliations:** 1 Institute of Physical Chemistry, Polish Academy of Sciences, Kasprzaka 44/52, 01-224 Warsaw, Poland; ksoltys@ichf.edu.pl (K.S.-B.); ks623@cam.ac.uk (K.S.); ijustyniak@ichf.edu.pl (I.J.); leszczynski.m@hotmail.com (M.K.L.); nolejnik@ichf.edu.pl (N.O.-F.); 2 Faculty of Chemistry, Warsaw University of Technology, Noakowskiego 3, 00-664 Warsaw, Poland

**Keywords:** organozinc complexes, coordination polymer, hydroxyquinoline, self-assembly

## Abstract

Introduction of photoactive building blocks into mixed-ligand coordination polymers appears to be a promising way to produce new advanced luminescent materials. However, rational design and self-assembly of the multi-component supramolecular systems is challenging from both a conceptual and synthetic perspective. Here, we report exploratory studies that investigate the potential of [Zn(q)_2_]_2_[*t*BuZn(OH)]_2_ complex (q = deprotonated 8-hydroxyquinoline) as an organozinc precursor as well as a mixed-ligand synthetic strategy for the preparation of new luminescent coordination polymers (CPs). As a result we present three new 2D mixed-ligand Zn(II)-quinolinate coordination polymers which are based on various zinc quinolinate secondary building units interconnected by two different organic linker types, i.e., deprotonated 4,4′-oxybisbenzoic acid (H_2_*obc*) as a flexible dicarboxylate linker and/or selected bipyridines (*bipy*). Remarkably, using the title organozinc precursors in a combination with H_2_*obc* and 4,4′-bipyridine, a novel molecular zinc quinolinate building unit, [Zn_4_(q)_6_(*bipy*)_2_(*obc*)_2_], was obtained which self-assembled into a chain-type hydrogen-bonded network. The application of the organometallic precursor allowed for its direct reaction with the selected ligands at ambient temperature, avoiding the use of both solvothermal conditions and additional base reagents. In turn, the reaction involving Zn(NO_3_)_2_, as a classical inorganic precursor, in a combination with H_2_*obc* and *bipy* led to a novel 1D coordination polymer [Zn_2_(q)_2_(NO_3_)_2_(*bipy*)]. While the presence of H_2_*obc* was essential for the formation of this coordination polymer, this ditopic linker was not incorporated into the isolated product, which indicates its templating behavior. The reported compounds were characterized by single-crystal and powder X-ray diffraction, elemental analysis as well as UV-Vis and photoluminescence spectroscopy.

## 1. Introduction

Coordination polymers (CPs), a class of crystalline porous materials made up of metal clusters or ions linked via organic ligands, represent a rapidly growing area in modern chemistry and materials science due to their wide range of applications [1,2,3,4,5]. In particular, luminescent CPs have attracted considerable attention in chemical sensing [6,7,8], biomedical imaging [9,10], nonlinear optics [11,12], as well as in light-emitting and display devices [13,14,15]. The tunable hybrid structure of CPs provides opportunities to manipulate their properties towards unusual luminescence behavior by varying the building blocks or structural connectivity [16,17,18]. Preparation of luminescent CPs has been achieved by several approaches, e.g., by application of photoactive metal cations, organic moieties, metal-organic charge transfer species or guest molecules [19,20,21]. One of the ligand systems known for its capability to form luminescent molecular complexes [22] and metal-organic materials [23] is 8-hydroxyquinoline (Hq) and its derivatives, which have been successfully applied in biomedicine [24] and the fabrication of optoelectronic devices such as organic light emitting diodes or solar cells [25,26]. Moreover, extended organic ligands based on the quinoline core have been applied in the development of luminescent coordination polymers, which represents an efficient strategy, albeit, often requiring multi-step preparation of organic ligand systems [27,28,29,30,31,32,33,34]. Alternatively, simple quinolinate ligands could be incorporated into mixed-ligand CPs as co-ligands, an approach which has the challenge of designing and controlling the self-assembly of multicomponent supramolecular systems [35], therefore it is not surprising that this approach has been explored to a lesser extent and only a few examples of well-defined functional quinolinate-based CPs [36,37,38], including materials with valuable luminescent properties [39,40], are reported.

In our previous reports we have demonstrated a series of novel molecular organozinc 8-hydroxyquinolinate complexes with high propensity to noncovalent interactions-driven self-organization into luminescent porous materials [41,42]. With this in mind and in continuation of our interest in both multifaceted organozinc chemistry [43,44,45] and the development of new synthetic approaches for the preparation of Zn-based functional materials [46,47,48,49,50,51], herein we report on the synthesis and characterization of a series of coordination polymers using a mixed-ligand synthetic strategy [35] and a [Zn(q)_2_]_2_[*t*BuZn(OH)]_2_ complex as a versatile organozinc precursor. The application of this well-defined organometallic precursor allowed for direct reaction with a ditopic organic acid and selected bipyridine ligands at ambient temperature, thus avoiding the use of both solvothermal conditions and additional base reagents. Using the mixed-ligand approach, we explore the influence of the metal precursor type and organic linker selection on both the molecular structure of quinolinate-based secondary building units (SBUs), their supramolecular architectures and the luminescence properties of the resulting CPs. We demonstrate the remarkable superiority of an organozinc precursor over the classic inorganic salt, regarding the structure-related luminescent properties of the resultant CPs.

## 2. Results and Discussion

### 2.1. Synthesis of Molecular Building Units and Coordination Polymers Based on Zn(II)-Quinolinate Species

**Scheme 1:**
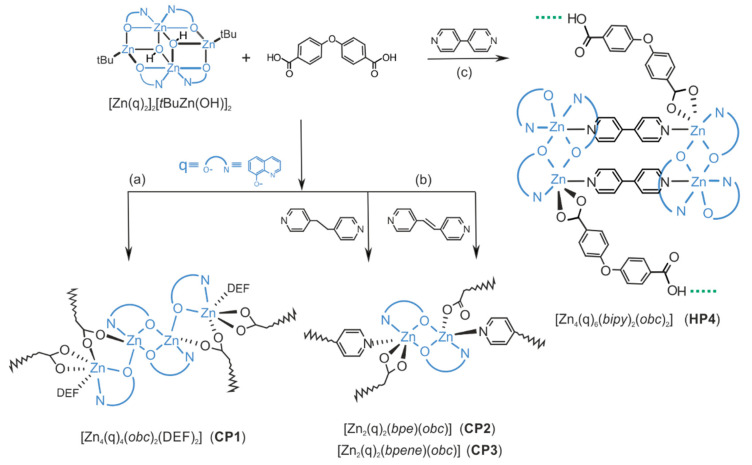
Representation of the synthetic pathways leading to luminescencent mixed-ligand Zn(II)-quinolinate coordination polymers: [Zn_4_(q)_4_(*obc*)_2_(DEF)_2_] (**a**); [Zn_2_(q)_2_(*bpe*)(*obc*)]*,* [Zn_2_(q)_2_(*bpene*)(*obc*)] (**b**) and molecular cluster [Zn_4_(q)_6_(*bipy*)_2_(*obc*)_2_] (**c**). Hydrogen bonds in **HP4** are marked with blue dashed lines.

Self-assembly of pre-designed functional molecular building units using mixed-ligand systems has arisen as a powerful tool for the development of novel porous materials due to the variety of supramolecular architectures possible [35]. In this regard, we selected the previously reported quinoline-based zinc complex [Zn(q)_2_]_2_[*t*BuZn(OH)]_2_ (q = deprotonated 8-hydroxyquinoline) [41,42] as a model luminescent core for exploratory studies investigating the potential of organozinc precursors and a mixed-ligand synthetic strategy for the preparation of new luminescent coordination polymers (CPs). In particular, these investigations concern the formation of CPs by self-assembly involving two different linker types: a flexible 4,4′-oxybisbenzoate linker and various bipyridine ligands (Scheme 1).

**Scheme 2:**
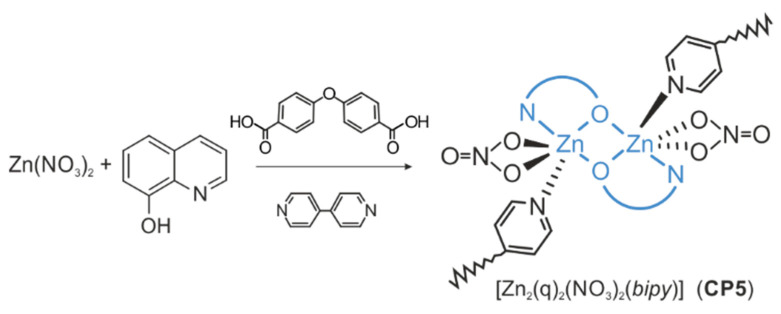
The inorganic synthetic approach to **CP5** with quenched luminescence.

Initially, a reference reaction involving only [Zn(q)_2_]_2_[*t*BuZn(OH)]_2_ and 4,4′-oxybisbenzoic acid (H_2_*obc*) in diethylformamide (DEF) was conducted, resulting in the formation of a 2D-coordination polymer [(Zn_4_(q)_4_(*obc*)_2_(DEF)_2_] (**CP1**), which is based on tetranuclear quinolinate-based SBU interconnected by the ditopic carboxylate linkers. (Scheme 1a). Moreover, two of the zinc centres in the **CP1** SBU are coordinated by solvent molecules, which suggests that introduction of additional neutral organic linkers could lead to new supramolecular architectures. Thus, in the next steps different bipyridne ligands were employed in the reaction with [Zn(q)_2_]_2_[*t*BuZn(OH)]_2_ and H_2_*obc* acid in order to evaluate the influence of the neutral ditopic linkers on the resulting CPs. In case of 1,2-bis(4-pyridyl)ethane (*bpe*), used as a flexible bipyridine ligand, a 2D coordination polymer [(Znq)_2_(*bpe*)(*obc*)] (**CP2**) was formed. This polymer incorporates dimeric [(Zn_2_(q)_2_] nodes interconnected by the ditopic *obc* and *bpe* linkers (Scheme 1b). When a more rigid 1,2-bis(4-pyridyl)ethylene (*bpene*) ligand was used, the reaction afforded a new 2D coordination polymer, [(Znq)_2_(*bpene*)(*obc*)] (**CP3**), containing the same dimeric SBU as **CP2**, but adopting different supramolecular architecture. Remarkably, using 4,4′-bipyridine (*bipy*) in the analogous reaction with [Zn(q)_2_]_2_[*t*BuZn(OH)]_2_ and H_2_*obc* led to completely different product: a molecular cluster [Zn_4_(q)_6_(*bipy*)_2_(*obc*)_2_] (**HP4**) self-assembled into chain-type hydrogen-bonded network (Scheme 1c). Finally, the applicability of zinc nitrate as an inorganic precursor in a combination with the same mixed organic ligand system was investigated. The reaction involving Zn(NO_3_)_2_, H_2_*obc* and *bipy* afforded a novel 1D coordination polymer [Zn_2_(q)_2_(NO_3_)_2_(*bipy*)] (**CP5**) (Scheme 2). While the presence of H_2_*obc* was essential for the product formation, this ditopic linker was not incorporated into the isolated product, which indicates its templating behaviour during the **CP5** formation. Strikingly, in contrast to luminescent zinc-quinolinate coordination polymers **CP1**, **CP2**, **CP3** and **HP4**, **CP5** was not luminescent (vide infra) despite involving the zinc quinolinate moieties; possibly on account of quenching behavior of nitrates (these aspects are a subject of our further ongoing investigations). The structures and composition of all of the resulting CPs were elucidated by means of elemental analysis and FTIR spectroscopy, single-crystal X-ray diffraction studies (SCXRD) and powder X-ray diffraction (PXRD), and they photophysical properties were tested using UV-Vis and photoluminescence (PL) spectroscopy. All the developed materials exhibited high stability upon prolonged storage (at least 3 months) under ambient conditions. Moreover, thermal stability of **CP1**, as a selected example, was investigated using thermogravimetric analysis (TGA/DTG), which revealed a mult-stage decomposition pathway with the first major step at ca. 193 °C (Figure S2).

### 2.2. Single Crystal X-ray Diffraction and Structure Analysis

**Figure 1:**
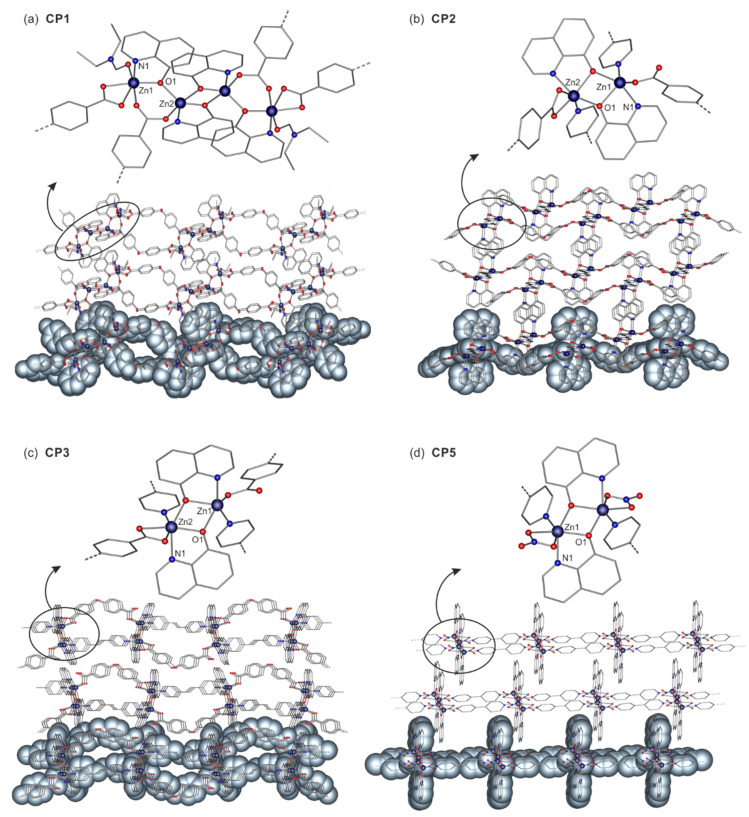
Molecular and supramolecular structures of **CP1** (**a**), **CP2** (**b**), **CP3** (**c**) and **CP5** (**d**). Zinc = violet, Oxygen = red, Nitrogen = blue, Carbon = grey. Most of the hydrogen atoms have been omitted for clarity.

According to the SCXRD analysis, **CP1** crystallizes in the monoclinic space group *P*2_1_/c. The tetranuclear SBU in **CP1** includes 5- and 6-coordinated zinc centres stabilized by the bridging quinolinate ligands as well as solvating DEF molecules connected to the terminal Zn atoms (Figure 1a). The extended supramolecular architecture of **CP1** involves connection of each of the SBU clusters with four bifunctional *obc* ligands, leading to the formation of 2D layers, which are arranged perpendicular to the crystallographic *a* axis and stacked in a staggered geometry with ca. 10.6 Å distance between adjacent layers (Figure 1a). The 2D framework of **CP1** exhibits the sql topology with Schläfli point symbol of {4^4^·6^2^} calculated for the clusters simplified as a 4-connecting node. Within each individual 2D layer of the polymer there are π···π interactions between the quinoline aromatic rings present in the central subunit [Zn_2_(q)_2_] of adjacent nodes. Moreover, the interactions between adjacent polymer layers are also based on the set of π···π contacts between the quinoline rings of the terminal fragments [Zn(q)(DEF)]. Interestingly, the molecular structure contains system of parallel 1D channels filled with solvent molecules. The channels are directed along the crystallographic *b* axis, parallel to the 2D layers in **CP1**. To provide the upper limit to space accessible to guest molecules in **CP1**, we have used procrystal electron density approach implemented in CrystalExplorer [52]. According to this calculations, **CP1** exhibit procrystal surface area of 1102 m^2^/g and the largest pore volumes of 543 Å^3^ per unit cell corresponding to 14% of the total material volume.

Compound **CP2** crystallizes in a monoclinic *P*2_1_/n space group. The dinuclear SBU in **CP2** comprises two Zn centres (5- and 6-coodrinated) stabilized by the quinolinate ligands as well as *obc* and *bpe* linkers (Figure 1b). The supramolecular arrangement of **CP2** involves the dinuclear SBU clusters, each connected by two pairs of bifunctional ligands: *obc* and *bpe*, leading to formation of extended 2D-layered structure directed perpendicular to the crystallographic *b* axis with a sql rhombic-grid topology, described with Schläfli symbol of {4^4^.6^2^} (Figure 1b). The supramolecular arrangement of the adjacent 2D layers can be described as an ABA sentence, with the interlayer distance about 10.8 Å, which is maintained by a set of cooperative noncovalent interactions. Moreover, the **CP2** structure includes a 2D system of pores filled with solvent molecules, extending along the *b* and *c* crystallographic axes of the structure. **CP2** has the procrystal surface area of about 2514 m^2^/g with pore volumes of 1494 Å^3^ (26% of total cell unit volume).

**CP3** crystallizes in the triclinic *P*-1 group and is composed of dinuclear SBUs interconnected by *obc* and *bpene* ligands (Figure 1c). The SBUs in **CP3** and **CP2** are structurally similar with the same connectivity of organic ligands, involving 5- and 6-coordinated zinc centers stabilized by quinolinate ligands. Interconnection of the dinuclear SBUs in **CP3** by the mixed-ligand systems leads to formation of extended 2D network layers with ellipsoidal openings approximately 11 × 21 Å in size. The adjacent polymer layers form a set of interlayer non-covalent interactions, which directs the layer stacking into the ABA sequence. The network topology on **CP3** is described with the point symbol {4^4^.6^2^}, which corresponds to the sql rhombic grid topology, similarly to **CP2**. However, the use of a more flexible *bpene* connector results in the adjacent layers forming more closely packed structure in **CP3**, compared to **CP2**. This, combined with the numerous non-covalent (C-H type) interactions between the individual polymer layers in **CP3**, creates a more compact 3D supramolecular structure. The supramolecular structure of **CP3** involves a system of 1D bottle-neck channels filled with solvent molecules, involving larger voids connected by narrower channels. The calculated procrystal surface area of **CP3** is 1754 m^2^/g and the cell consist of 20% (505 Å^3^) of free volume.

**Figure 2:**
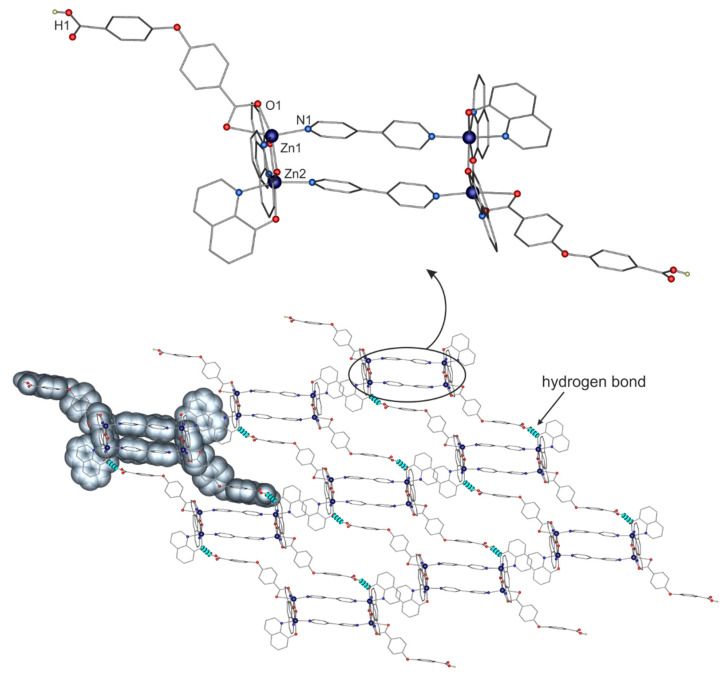
Molecular and supramolecular structure of **HP4**; zinc = violet, oxygen = red, nitrogen = blue, carbon = grey. Hydrogen bonds are marked with blue dashed lines. Hydrogen atoms have been omitted for clarity.

Compound **HP4**, with the chemical formula [Zn_4_(q)_6_(*bipy*)_2_(H*obca*)_2_], crystallizes in the monoclinic *C*2/c space group and exhibits clear differences in comparison to the **CP1**–**CP3** in the character of molecular and supramolecular structure (Figure 2). The molecular cluster [Zn_4_(q)_6_(*bipy*)_2_(H*obca*)_2_] exhibits a macrocyclic structure with two [Zn_2_(q)_3_(H*obc*)] interconnected by two bipyridine ligands (Figure 2). The supramolecular structure of **HP4** is governed through cooperative O-H···O hydrogen interactions and π-π interactions. The molecular units of **HP4** self-assemble via cooperative O-H···O hydrogen interactions (with the O···O distance of 2.469 Å) mediated by the distal carboxylic groups and affording a 1D chain-type structure. Additionally, the 1D chains are self-organized by π-π interactions of the quinolinate aromatic ring into a 2D supramolecular system with open channels extending in the direction perpendicular to the crystallo-graphic c axis and occupied by solvent molecules. **HP4** exhibits procrystal surface area of 1902 m^2^/g and the largest pore volumes of 2611 Å^3^ per unit cell corresponding to 22% of the total material volume.

As evidenced by the SCXRD, compound **CP5** (space group *P*-1) is a 1D coordination polymer formed as a result of the bridging of each dimeric node [(Zn_2_(q)_2_(NO_3_)_2_] by two linear *bipy* ligands (Figure 1d). The [(Znq)_2_(NO_3_)_2_] SBU in **CP5** contains the [Zn_2_(q)_2_] core involving two zinc centres with octahedral geometry, each coordinated by a nitrate group. Analysis of the supramolecular structure of **CP5** revealed that the adjacent polymer chains are of the distance 2.377 Å between oxygen of the nitrate group and hydrogen of the pyridyl ring of *bipy* of adjacent polymer chain. Thus, defined 2D adjacent layers of **CP5**, stacked together by CH···π interactions (2.830 Å) between hydrogen of quinoline ligand and electrons of pyridyl ring of *bipy* and by π···π interactions (3.371 Å) between quinoline rings create 3D supramolecular architecture with slip along the crystallographic *b* axis. Furthermore, packing of the layers generates a 2D pore system filled with solvent molecules within the crystal structure. The calculated procrystal surface area of **CP5** is 1585 m^2^/g and pore volume is 172 Å^3^ (19% of the total volume of the unit cell).

We also note that to evaluate the phase purity of the developed products, powder X-ray diffraction (PXRD) experiments were conducted. For all products the experimental PXRD patterns displayed characteristics closely corresponding to the reference patterns simulated from the collected single crystal data (Figure S1), while some minor differences could be attributed to the different measurement temperatures as well as texturing of powder samples.

### 2.3. FTIR Spectroscopic Study

In order to further investigate the coordination modes of carboxylate and quinolinate moieties in compounds **CP1**–**CP5** and **HP4** their FTIR spectra in solid state (Figures S3–S7) were analysed and compared to the spectra of selected reference compounds: [Zn(q)_2_]_2_[*t*BuZn(OH)]_2_ precursor, Hq ligand and H_2_*obc* linker (Figures S8–S10) as well as the literature references [53,54]. As a result, we found that the bands related to the quinolinate ligand (most characteristic bands observed in the 1580–1322 cm^−1^ and 825–730 cm^−1^ ranges) in the spectra of **CP1**–**CP5** and **HP4** were shifted to the higher frequencies in comparison to the free Hq ligand, which is in agreement with the spectrum of the [Zn(q)_2_]_2_[*t*BuZn(OH)]_2_ precursor [41,42]. Moreover, the spectrum of **CP1** contains a sharp high intensity band at 1379 cm^−1^ corresponding to the symmetric vibrations of the carboxyl group and two bands at 1598, cm^−1^ representing symmetric vibrations of the carboxylate group. These results indicate presence of two different bidentate modes of the carboxylate binding in the structure of **CP1**. The asymmetric and symmetric stretching vibrational modes of the carboxylate ligand for both complexes **CP2** and **CP3** are further split into two peaks, respectively: 1615, 1597 cm^−1^ and 1381, 1367 cm^−1^ (in **CP2**); and 1600, 1578 cm^−1^ and 1378, 1363 cm^−1^ (in **CP3**). These observations confirm the existence of monodentate and chelating carboxylate group in the structures of **CP2** and **CP3**. The absence of band at ~1700 cm^−1^ for **CP1**–**CP3** compounds confirm complete deprotonation of carboxyl groups. For compound **HP4** the asymmetric and symmetric vibrations of the carboxylate species were observed at 1595 and 1381 cm^−1^, respectively, while a band at 1694 cm^−1^ corresponds to the stretching vibrations of C=O carbonyl group in **HP4**. The nitrate ion stretching vibration bands for **CP5** were found at 1464 and 1298 cm^−1^. The separation of 166 cm^−1^ between them is consistent with a bidentate coordination of the nitrate ion. Stretching vibrations of the carbonyl group from DEF molecules coordinated to the Zn center in **CP1** gives band at 1634 cm^−1^, while the guest molecules located in the free space of the structure exhibit the band at 1670 cm^−1^. The solvent guest DMF molecules in **CP2**–**CP5** and **HP4** are represented by stretching C=O bands at 1666–1672 cm^−1^. Importantly, the observation of the characteristic bands related to the carboxylate moieties in **CP1**–**CP3** and **HP4** well corroborate with the coordination modes of carboxylate ligands judged from the X-ray diffraction study.

### 2.4. Photophysical Characterization

**Figure 3:**
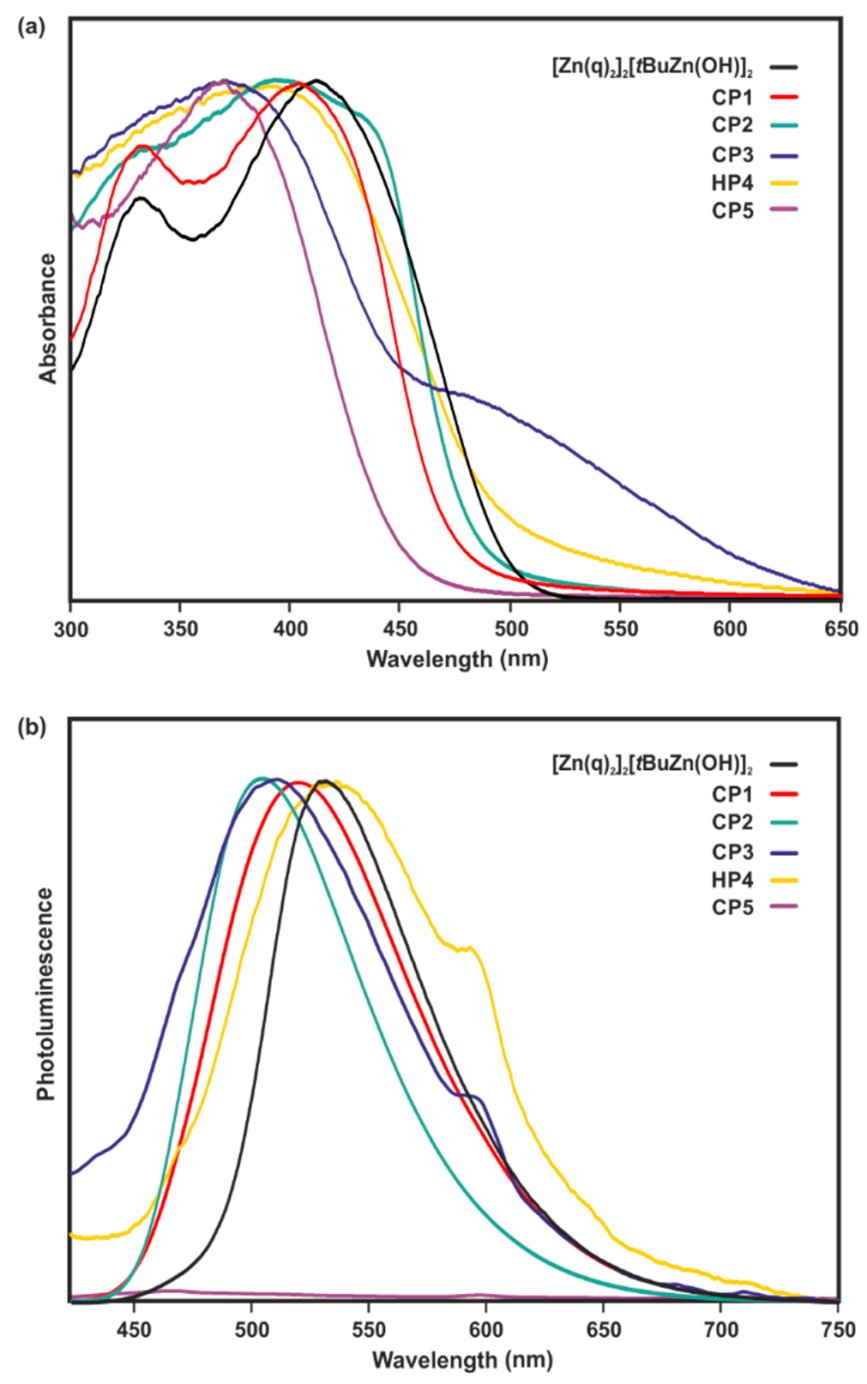
Normalized (**a**) UV-Vis absorption measured in diffusive reflectance of compound powders dispersed with BaSO_4_, (**b**) luminescent spectra measured in the solid-state of the [Zn(q)_2_]_2_[*t*BuZn(OH)]_2_ precursor and **CP1**–**CP5** and **HP4** coordination polymers at room temperature; excitation at 400 nm.

Solid-state UV-Vis spectra of the developed materials were measured in diffusive reflectance geometry and transformed using Kubelka-Munk equation. As shown in Figure 3a and Figure S11, UV-Vis absorption maxima appear at 404 nm for **CP1**, 393 nm for **CP2**, 371 nm for **CP3**, 392 nm for **HP4**, and 370 nm for **CP5**, showing blue shifts compared to that observed for the [Zn(q)_2_]_2_[*t*BuZn(OH)]_2_ precursor (absorption maximum at 410 nm). It should be noted that the solid-state UV-Vis absorption spectrum of **CP3** exhibited long tail extending through visible region possibly due to the scattering effect. Under the same excitation conditions (λ_ex_: 400 nm), **CP1**–**CP3** and **HP4** polymers exhibit intense photoluminescence with the emission maximum: 520 nm (**CP1**), 505 nm (**CP2**), 511 nm (with a shoulder peak at ~590 nm) (**CP3**) and 537 nm (with a shoulder peak at ~590 nm) (**HP4**) (Figure 3b). In turn, **CP5** does not exhibit luminescent properties. The maxima of the absorption bands of the **CP1**, **CP2**, and **CP3** polymers are blue-shifted, while the emission band of **HP4** is red-shifted, in comparison to that observed for [Zn(q)_2_]_2_[*t*BuZn(OH)]_2_ (emission maximum at 530 nm). The observed photoluminescence maxima for these coordination polymers are also blue-shifted compared to other organozinc 8-hydroxyquinolinate complexes: [*t*BuZn(q)]_3_ (emission band at 555 nm for λ_ex_ = 350 nm) and [(*t*Bu)_3_Zn_5_(µ_4_-O)(q)_5_] (emission band at 535 nm for λ_ex_ = 350 nm) [41]. The emission maxima of **CP1**, **CP2** and **CP3** are also different from the PL of the well-studied compound Zn(q)_2_ [55] (536 nm for λ_ex_ = 400 nm), being blues-hifted by 16, 31 and 25 nm, respectively. Maximum of the **HP4** emission spectra is comparable with that for Zn(q)_2_. In turn, the emission maxima of **CP1**, **CP3** and **HP4** are red-shifted in comparison to emission maximum of α-Al(q)_3_ crystal phase (504 nm for λ_ex_ = 365 nm) while the emission maximum of **CP2** is comparable [56]. The resulting spectra clearly indicate that the introduction of various bipyridine ligands allows for tailoring both the absorption and emission properties of the developed materials. Moreover, the data lead to the conclusion that increase of the coordination framework rigidity through crosslinking of metal centers with dicarboxylate and bipyridine ligands affects blueshift of the emission maximum within this class of materials. Surprisingly, the **CP5** exhibits no luminescence, despite its great structural similarities to the other studied luminescent materials (**CP1**–**CP3** and **HP4**), which could be related to the quenching effect of the inorganic nitrate ligands present in the structure of **CP5**. This intriguing issue is a subject of our ongoing investigations which will be published due course.

## 3. Conclusions

The multifaceted chemistry of molecular organometallic complexes incorporating bidentate quinolinate ligands as potential precursors of functional materials has remained a largely undeveloped area of research, which significantly hampers the possibility of obtaining novel fluorescent hybrid materials. Herein, we demonstrate a new synthetic approach to photoluminescent quinolinate ligand-based CPs that allowed for the isolation of a series of structurally diverse hybrid organic-inorganic materials. The application of well-defined organozinc precursor allowed for direct reaction with a ditopic organic acid and a selected bipyridine ligand at ambient temperature, avoiding the use of both solvothermal conditions and additional base reagents. Using the mixed-ligand approach we explored the influence of the metal precursor type and organic linker selection on both the molecular structure of quinolinate-based SBUs and supramolecular architectures alongside luminescence properties of the resulting CPs. The presented approach appears as an intriguing strategy for the design and construction of new luminescent materials based on 8-hydroxyquinoline derivatives. We believe that the reported synthetic strategy unveils new prospects for the design of reaction systems leading to a variety of novel luminescent CP networks of interest in sensing, optoelectronics, and (photo) catalysis.

## 4. Materials and Methods

### 4.1. General Information

All chemical reagents, including 8-hydroxyquinoline (Hq) and 4,4′-oxybisbenzoic acid (H_2_*obc*), 1,2-bis(4-pyridyl)ethane (*bpe*), 1,2-bis(4-pyridyl)ethylene (*bpene*), 4,4′-bipyridine (*bipy*) and Zn(q)_2_ were purchased from commercial vendors. Solvents were dried and distilled prior to use. All of the described synthetic procedures were carried out under a nitrogen atmosphere using standard Schlenk and glovebox techniques. Synthesis of **CP5** was performed under an air atmosphere. The [Zn(q)_2_]_2_[*t*BuZn(OH)]_2_ precursor was obtained according to our previously reported procedure [41]. The reported synthetic procedures demonstrate the most efficient ways (in terms of yield and product purity) for preparation of the discussed products, that we were able to find during the study.

Fourier Transform Infrared Attenuated Total Reflectance (FTIR ATR) were recorded (500–4000 cm^−1^ region) on a Tensor instrument (Bruker, Billerica, MA, USA) equipped with an ATR accessory. Powder X-ray diffraction (PXRD) data were collected on Empyrean diffractometer (PANalytical, Almelo, Netherlands). Measurements employed Ni-filtered Cu Kα radiation of a copper sealed tube charged with 40 kV voltage and 40 mA current and Bragg Brentano geometry and a Si zero-background holder. Diffraction patterns were measured in the range of 2θ = 3−60° of scattering angle by step scanning with step of 0.02°. Thermogravimetric and derivative thermogravimetric analyses (TGA/DTG) were recorded on Q600 apparatus (TA Instruments, Milford, MA, USA). Samples for thermogravimetric characterization was placed in alumina crucibles under an argon atmosphere at heating rate of 5 °C/min. Optical absorption (UV-Vis) measurements were carried out using a UV-2600 spectrophotometer (Shimadzu, Kyoto, Japan). Photoluminescence (PL) spectra were recorded on F-7000 fluorescence spectrophotometer (Hitachi, Tokyo, Japan).

### 4.2. Synthesis of [(Zn_4_(q)_4_(obc)_2_(DEF)_2_] (**CP1**)

A THF (5 mL) solution of [Zn(q)_2_]_2_[tBuZn(OH)]_2_ (0.090 mg, 0.091 mmol) was cooled to −78 °C and H_2_obc (0.024 g, 0.091 mmol) in THF (5 mL) was added using a syringe. The reaction mixture was stirred for 15 min to warm it up to room temperature. The solid residue obtained after removal of the solvent under reduced pressure using a high vacuum pump, was recrystallized from DEF (5 mL). The crystallization overnight at 4 °C resulted in formation yellow crystals of polymer **CP1**. The crystal product was decanted and the solution was removed using a syringe. Then solid product was washed with DEF (3 mL) and CH_2_Cl_2_ (3 mL). The washed product was dried at room temperature using vacuum pump at 10^−3^ mbar for 3 h. Isolated yield: 72%. IR (ATR): *ṽ* [cm^−1^] = 1670 (m), 1634 (m), 1598 (s), 1579 (m), 1556 (m), 1496 (s), 1467 (s), 1379 (s), 1322 (s), 1278 (m), 1261 (m), 1236 (s), 1159 (m), 1126 (w), 1106 (s), 1035 (w), 1012 (m), 959 (w), 944 (w), 908 (w), 876 (m), 863 (w), 824 (s), 804 (m), 782 (s), 770 (m), 750 (m), 735 (s), 709 (w), 696 (w), 658 (m), 643 (w), 626 (m), 607 (w), 593 (w), 518 (w), 503 (m), 421 (m). Elemental analysis (%) calcd for [C_74_H_60_N_6_O_16_Zn_4_·2(C_5_H_11_NO)]: C 57.55, H 4.71, N 6.39; found C 57.25, H 4.67, N 6.48.

### 4.3. Synthesis of [(Znq)_2_(bpe)(obc)] (**CP2**)

A DMF (12 mL) solution of [Zn(q)_2_]_2_[*t*BuZn(OH)]_2_ (0.030 g, 0.030 mmol) was cooled to −78 °C and mixture of H_2_*obc* (0.016 g, 0.061 mmol) and *bpe* (0.011 g, 0.061 mmol) in DMF (4 mL) was added using a syringe. The reaction mixture was stirred for 15 min to warm to room temperature and the resultant mixture was left overnight at 4 °C. The resulting precipitate was removed by filtration using a syringe filter (pore size 0.45 µm) and then the solution was concentrated to ~10 mL at a vacuum pump for ~2 h. Overnight crystallization at room temperature resulted in the formation of yellow crystals of polymer **CP2**. The crystal product was decanted and the solution was removed using a syringe. The product was washed three times with DMF (3 mL). The washed product was dried at room temperature using a vacuum pump (10^−3^ mbar) for 3 h. Isolated yield: 58%. IR (ATR): *ṽ* [cm^−1^] = 1672 (s), 1615 (m), 1597 (s), 1573 (m), 1549 (m), 1494 (s), 1462 (s), 1434 (m), 1406 (m), 1381 (s), 1367 (s), 1323 (s), 1279 (m), 1228 (s), 1160 (m), 1135 (w), 1108 (s), 1089 (s), 1073 (m), 1062 (m), 1028 (m), 1011 (w), 907 (w), 874 (m), 824 (s), 803 (m), 784 (s), 768 (m), 731 (s), 713 (w), 697 (w), 659 (m), 648 (m), 632 (w), 618 (w), 606 (w), 579 (w), 554 (w), 540 (w), 500 (m), 440 (w). Elemental analysis (%) calcd for [C_44_H_32_N_4_O_7_Zn_2_·3(C_3_H_7_NO)]: C 58.98, H 5.00, N 9.08; found C 58.82, H 4.94, N 9.17.

### 4.4. Synthesis of [(Znq)_2_(bpene)(obc)] (**CP3**)

A DMF (4 mL) solution of [Zn(q)_2_]_2_[*t*BuZn(OH)]_2_ (0.040 g, 0.041 mmol) was cooled to −78 °C and H_2_*obc* (0.005 g, 0.020 mmol) in DMF (3 mL) and *bpene* (0.002 g, 0.010 mmol) in DMF (3 mL) was added using a syringe. The reaction mixture was then stirred for 15 min while warming to room temperature. Crystallization at room temperature resulted in the formation of yellow crystals of polymer **CP3** after three days. The crystalline product was decanted and the solution was removed using a syringe. The product was washed three times with DMF (3 mL). The washed product was dried for 3 h at room temperature using a high-vacuum pump (10^−3^ mbar). Isolated yield: 64%. IR (ATR): *ṽ* [cm^−1^] = 1668 (m), 1634 (m), 1600 (s), 1578 (s), 1556 (m), 1497 (s), 1466 (s), 1378 (s), 1363 (m), 1322 (s), 1277 (m), 1236 (s), 1214 (m), 1159 (m), 1138 (w), 1106 (s), 1068 (w), 1035 (w), 1011 (w), 945 (w), 908 (w), 877 (m), 864 (w), 825 (s), 804 (m), 783 (s), 769 (m), 747 (m), 734 (s), 696 (w), 658 (m), 644 (w), 626 (w), 596 (w), 568 (w), 504 (m), 491 (m). Elemental analysis (%) calcd for [C_44_H_30_N_4_O_7_Zn_2_·(C_3_H_7_NO)]: C 60.66, H 4.01, N 7.53; found C 60.45, H 3.96, N 7.68.

### 4.5. Synthesis of the Molecular Cluster [Zn_4_(q)_6_(bipy)_2_(obc)_2_] (**HP4**)

A DMF (8 mL) solution of [Zn(q)_2_]_2_[*t*BuZn(OH)]_2_ (0.090 g, 0.091 mmol) was cooled to −78 °C and mixture of H_2_*obc* (0.047 g, 0.182 mmol) and *bipy* (0.028 g, 0.182 mmol) in DMF (4 mL) was added using a syringe. The reaction mixture was stirred for 10 min while it warmed to room temperature and the resultant suspension was dissolved by addition of DMF (8 mL) and stirred at 60 °C for a few minutes until the precipitate dissolved. The obtained solution was filtered using a syringe filter (pore size 0.45 µm) and concentrated to ~7 mL. The crystallization at 4 °C resulted in the formation of yellow crystals of compound **HP4** after two days. The crystalline product was decanted and the solution was removed using a syringe. The product was washed three times with DMF (3 mL). The washed product was dried at room temperature using vacuum pump at 10^−3^ mbar for 2 h. Isolated yield: 52%. IR (ATR): *ṽ* [cm^−1^] = 1666 (m), 1595 (m), 1571 (m), 1535 (w), 1495 (s), 1460 (s), 1407 (w), 1381 (s), 1324 (s), 1280 (m), 1235 (s), 1218 (s), 1176 (w), 1159 (m), 1108 (s), 1096 (m), 1065 (m), 1045 (w), 1032 (m), 1010 (m), 990 (w), 964 (w), 906 (w), 876 (m), 856 (w), 824 (m), 803 (s), 788 (s), 748 (m), 730 (s), 694 (m), 674 (w), 648 (m), 632 (m), 605 (m), 584 (m), 514 (m), 495 (s), 454 (m). Elemental analysis (%) calcd for [C_102_H_70_N_10_O_16_Zn_4_·2(C_3_H_7_NO)]: C 61.79, H 4.03, N 8.01; found C 61.65, H 3.97, N 8.09.

### 4.6. Synthesis of [Zn_2_(q)_2_(NO_3_)_2_(bipy)] (**CP5**)

Hq (0.100 g, 0,689 mmol) and Zn(NO_3_)_2_·6H_2_O (0.205 g, 0.689 mmol) were dissolved in DMF (3 mL) and the reaction mixture was stirred for few minutes at room temperature, then the mixture of H_2_*obc* (0.089 g, 0.344 mmol) and *bipy* (0.054 g, 0.344 mmol) in DMF (3 mL) was added using syringe. The crystallization at room temperature resulted in formation yellow crystals of compound **CP5** after two days. The crystal product was decanted and the solution was removed using a syringe. The product was washed three times with DMF (2 mL). The washed product was dried at room temperature using vacuum pump at 10^−3^ mbar for 1 h. Isolated yield: 46%. IR (ATR): *ṽ* [cm^−1^] = 1666 (m), 1609 (m), 1580 (m), 1538 (w), 1495 (m), 1464 (s), 1418 (m), 1382 (s), 1322 (s), 1298 (s), 1276 (s), 1242 (m), 1229 (m), 1177 (w), 1136 (w), 1108 (s), 1077 (m), 1048 (w), 1031 (m), 1013 (m), 980 (w), 962 (w), 911 (w), 869 (w), 822 (s), 802 (m), 787 (m), 747 (m), 733 (s), 661 (m), 639 (s), 608 (m), 594 (m), 573 (m), 510 (m), 501 (s), 468 (m). Elemental analysis (%) calcd for [C_28_H_20_N_6_O_8_Zn_2_·(C_3_H_7_NO)]: C 48.21, H 3.52, N 12.70; found C 48.08, H 3.47, N 12.83.

### 4.7. Single-Crystal X-ray Diffraction Studies

The crystals were selected under Paratone-N oil, mounted on nylon loops and positioned in the cold stream of the diffractometer. The X-ray data for complex **CP1** were collected on a Nonius Kappa CCD diffractometer (Billerica, MA, USA) using graphite monochromated MoKα radiation (λ = 0.71073 Å). The data were processed with DENZO and SCALEPACK (HKL2000 package) [57]. The X-ray data for complexes **CP2**, **CP3**, **HP4**, and **CP5** were collected at 100(2) K on a SuperNova diffractometer (Agilent, Santa Clara, CA, USA) using Mo*Kα* radiation (λ = 0.71073 Å). The data were processed with *CrysAlisPro* [58]. Structures were solved by direct methods and refined using *SHELXL-2016/4* [59]. All non-hydrogen atoms were refined with anisotropic displacement parameters. Hydrogen atoms were added to the structure model at geometrically idealized coordinates and refined as riding atoms.

For compounds **CP2**, **CP3** and **HP4** we observed significant residual electron densities within the pores from the solvent molecule. The solvent molecules appeared to be highly disordered and it was difficult to reliably model their positions and distribution, therefore, the *SQUEEZE* function of *PLATON* [60] was used to eliminate the contribution of the electron density in the solvent region from the intensity data. See Supporting Information for more details on the SCXRD experiments and data refinement.

## Data Availability

The data presented in this study are available from the supplementary materials.

## Supplementary Materials

The following are available online, Table S1: Crystal data and structure refinement details for **CP1**, Table S2: Crystal data and structure refinement details for **CP2**, Table S3: Crystal data and structure refinement details for **CP3**, Table S4: Crystal data and structure refinement details for **HP4**, Table S4: Crystal data and structure refinement details for **CP5**, Figure S1: Experimental and simulated PXRD patterns for the **CP1**-**CP5** and **HP4** polymers, Figure S2: Thermogravimetric and derivative thermogravimetric profiles (TGA/DTG) of the as-synthesized sample of **CP1**. Figures S3–S7: FT-IR spectrum of the **CP1**-**CP5** and **HP4** compounds. Figures S8–S10: FT-IR spectrum of the [Zn(q)_2_]_2_[*t*BuZn(OH)]_2_, Hq and H_2_*obc*. Figure S11: UV-Vis absorption and the photoluminescence solid-state spectra of polymers: (a) **CP1**, (b) **CP2**, (c) **CP3**, (d) **HP4**. CCDC 2092607−2092611 contain the supplementary crystallographic data for this paper. These data can be obtained free of charge via www.ccdc.cam.ac.uk/data_request/cif, or by emailing data_request@ccdc.cam.ac.uk, or by contacting The Cambridge Crystallographic Data Centre, 12 Union Road, Cambridge CB2 1EZ, UK; Fax: +44-1223-336033.

## Sample Availability

Samples of the compounds are not available from the authors.

## References

[1] Li J.R., Sculley J., Zhou H.C. (2012). Metal-Organic Frameworks for Separations. Chem. Rev..

[2] Zhu L., Liu X., Jiang H., Sun L. (2017). Metal−Organic Frameworks for Heterogeneous Basic Catalysis. Chem. Rev..

[3] Edem B., Huang J., Zeng J., Subhan F., Feng F., Zhang Y., Qiu Z., Aslam S., Li G., Yan Z. (2020). Magnetic Metal–Organic Framework Composites for Environmental Monitoring and Remediation. Coord. Chem. Rev..

[4] Connolly B.M., Mehta J.P., Moghadam P.Z., Wheatley A.E.H., Fairen-Jimenez D. (2018). From Synthesis to Applications: Metal–Organic Frameworks for an Environmentally Sustainable Future. Curr. Opin. Green Sustain. Chem..

[5] Qiu Q., Chen H., Wang Y., Ying Y. (2019). Recent Advances in the Rational Synthesis and Sensing Applications of Metal-Organic Framework Biocomposites. Coord. Chem. Rev..

[6] Cui Y., Yue Y., Qian G., Chen B. (2012). Luminescent Functional Metal-Organic Frameworks. Chem. Rev..

[7] Li S.J., Ghosh S.K., Lustig W.P., Desai A. (2017). V Metal-Organic Frameworks: Functional Luminescent and Photonic Materials for Sensing Applications. Chem. Soc. Rev..

[8] Liu J., Luo Z., Pan Y., Kumar A., Trivedi M., Kumar A. (2020). Recent Developments in Luminescent Coordination Polymers: Designing Strategies, Sensing Application and Theoretical Evidences. Coord. Chem. Rev..

[9] Zhao H., Zou Q., Sun S., Yu C., Zhang X., Li R., Fu Y. (2016). Theranostic Metal-Organic Framework Core-Shell Composites for Magnetic Resonance Imaging and Drug Delivery. Chem. Sci..

[10] Wang H.-S. (2017). Metal–Organic Frameworks for Biosensing and Bioimaging Applications. Coord. Chem. Rev..

[11] Liu Y., Li G., Li X., Cui Y. (2007). Cation-Dependent Nonlinear Optical Behavior in an Octupolar 3D Anionic Metal-Organic Framework. Angew. Chem. Int. Ed..

[12] Huang X., Li Q., Xiao X., Jia S., Li Y., Duan Z., Bai L., Yuan Z. (2018). Nonlinear-Optical Behaviors of a Chiral Metal−Organic Framework Comprised of an Unusual Multioriented Double-Helix Structure. Inorg. Chem..

[13] Stavila V., Talin A.A., Allendorf M.D. (2014). MOF-Based Electronic and Optoelectronic Devices. Chem. Soc. Rev..

[14] Goswami S., Chen M., Wasielewski M.R., Farha O.K., Hupp J.T. (2018). Boosting Transport Distances for Molecular Excitons within Photoexcited Metal−Organic Framework Films. ACS Appl. Nano Mater..

[15] Lustig W.P., Li J. (2018). Luminescent Metal–Organic Frameworks and Coordination Polymers as Alternative Phosphors for Energy Efficient Lighting Devices. Coord. Chem. Rev..

[16] Wu Z., Tan B., Wang J., Du C., Deng Z. (2015). Tunable Photoluminescence and Direct White-Light Emission in Mg-Based Coordination Networks. Chem. Commun..

[17] Jensen S., Tan K., Lustig W.P., Kilin D.S., Li J., Chabal Y.J., Thonhauser T. (2019). Structure-Driven Photoluminescence Enhancement in a Zn-Based Metal−Organic Framework. Chem. Mater..

[18] Li J., Yuan S., Qin J., Pang J., Zhang P., Zhang Y., Huang Y., Drake H.F., Liu W.R., Zhou H. (2020). Stepwise Assembly of Turn-on Fluorescence Sensors in Multicomponent Metal–Organic Frameworks for In Vitro Cyanide. Angew. Chem. Int. Ed..

[19] Allendorf M.D., Bauer C.A., Bhakta R.K., Houk R.J.T. (2009). Luminescent Metal–Organic Frameworks. Chem. Soc. Rev..

[20] Heine J., Muller-Buschbaum K. (2013). Engineering Metal-Based Luminescence in Coordination Polymers and Metal–Organic Frameworks. Chem. Soc. Rev..

[21] Karmakar A., Samanta P., Desai A.V., Ghosh S.K. (2017). Guest-Responsive Metal−Organic Frameworks as Scaffolds for Separation and Sensing Applications. Acc. Chem. Res..

[22] Phillips J.P. (1956). The Reaction of 8-Quinolinol. Chem. Rev..

[23] Albrecht M., Fiege M., Osetska O. (2008). 8-Hydroxyquinolines in Metallosupramolecular Chemistry. Coord. Chem. Rev..

[24] Prachayasittikul V., Prachayasittikul S., Ruchirawat S., Prachayasittikul V. (2013). 8-Hydroxyquinolines: A Review of Their Metal Chelating Properties and Medicinal Applications. Drug Des. Devel. Ther..

[25] Tang C.W., Vanslyke S.A. (1987). Organic Electroluminescent Diodes. Appl. Phys. Lett..

[26] Kaji H., Suzuki H., Fukushima T., Shizu K., Suzuki K., Kubo S., Komino T., Oiwa H., Suzuki F., Wakamiya A. (2015). Purely Organic Electroluminescent Material Realizing 100% Conversion from Electricity to Light. Nat. Commun..

[27] Sun Q., Yang K., Ma W., Zhang L., Yuan G. (2020). A Highly STable 8-Hydroxyquinolinate-Based Metal–Organic Framework as a Selective Fluorescence Sensor for Fe^3+^, Cr_2_O_7_^2−^ and Nitroaromatic Explosives. Inorg. Chem. Front..

[28] Zhang L., Sun L., Li X., Tian Y., Yuan G. (2015). Five 8-Hydroxyquinolinate-Based Coordination Polymers with Tunable Structures and Photoluminescent Properties for Sensing Nitroaromatics. Dalton Trans..

[29] Zhang L., Rong L., Hu G., Jin S., Jia W., Liu J., Yuan G. (2015). Six Zn (II) and Cd (II) Coordination Polymers Assembled from a Similar Binuclear Building Unit: Tunable Structures and Luminescence Properties. Dalton Trans..

[30] Zhao X., Wang S., Zhang L., Liu S., Yuan G. (2018). 8-Hydroxyquinolinate-Based Metal-Organic Frameworks: Synthesis, Tunable Luminescent Properties, and Highly Sensitive Detection of Small Molecules and Metal Ions. Inorg. Chem..

[31] Yuan G., Huo Y., Nie X., Jiang H., Liu B., Fang X., Zhao F. (2013). Controllable Supramolecular Structures and Luminescent Properties of Unique Trimeric Zn(II) 8-Hydroxyquinolinates Tuned by Functional Substituents. Dalton Trans..

[32] Yuan G., Zhang Q., Wang Z., Song K., Yuan X., Wang Y., Zhang L. (2017). Assembly of Four 8-Quinolinate-Based Multinuclear Complexes: The Effect of Substituents on Core Structures and Photoluminescent Properties. Inorg. Chem. Front..

[33] Yuan G., Shan W., Xuelong Q., Ma L., Huo Y. (2014). Self-Assembly of Five 8-Hydroxyquinolinate-Based Complexes: Tunable Core, Supramolecular Structure and Photoluminescence Properties. Chem. Asian J..

[34] Yuan G., Hu G., Shan W., Jin S., Gu Q., Chen J. (2015). Structural and Luminescent Modulation in 8-Hydroxyquinolinate-Based Coordination Polymers by Varying Dicarboxylic Acids. Dalton Trans..

[35] Du M., Li C.-P., Liu C.-S., Fang S.-M. (2013). Design and Construction of Coordination Polymers with Mixed-Ligand Synthetic Strategy. Coord. Chem. Rev..

[36] De Carvalho A.B., de Souza Í.P., de Andrade L.M., Binatti I., Pedroso E.F., Krambrock K., Oliveira W.X.C., Pereira-Maia E.C., Silva-Caldeira P.P. (2018). Novel Copper (II) Coordination Polymer Containing the Drugs Nalidixic Acid and 8-Hydroxyquinoline: Evaluation of the Structural, Magnetic, Electronic and Antitumor Properties. Polyhedron.

[37] Xiang J., Jia L., Man W., Qian K., Yiu S., Lee G., Peng S.-M., Gao S., Lau T.-C. (2011). Novel Heterobimetallic Ruthenium(III)–cobalt(II) Compounds Constructed from Trans-[RuIII(Q)_2_(CN)_2_]^−^ (Q = 8-Quinolinolato): Synthesis, Structures and Magnetic Propertiesw. Chem. Commun..

[38] Montes V.A., Zyryanov G.V., Danilov E., Agarwal N., Palacios M.A., Anzenbacher P. (2009). Ultrafast Energy Transfer in Oligofluorene-Aluminum Bis(8-Hydroxyquinoline) Acetylacetone Coordination Polymers. J. Am. Chem. Soc..

[39] Wang Y.J., Li H.H., Chen Z.R., Huang C.C., Huang X.H., Feng M., Lin Y. (2008). A Series of Lead (II)/Iodine Hybrid Polymers Based on 1-D and 2-D Metal–Organic Motifs Linked by Different Organic Conjugated Ligands. Cryst. Eng. Comm..

[40] Guochun Z., Chengfang Q., Jiehui L., Qing W., Zhengqiang X., Sanping C. (2015). Hydrothermal Synthesis, Structure and Property of Transition Metal (Mn, Zn, Cd or Pb) Coordination Frameworks Using Quinoline-8-Oxy-Acetate Acid and Dicarboxylic Acid as Ligands. Chem. Res. Chin. Univ..

[41] Sokołowski K., Justyniak I., Śliwiński W., Sołtys K., Tulewicz A., Kornowicz A., Moszyński R., Lipkowski J., Lewiński J. (2012). Towards a New Family of Photoluminescent Organozinc 8-Hydroxyquinolinates with a High Propensity to Form Noncovalent Porous Materials. Chem. Eur. J..

[42] Sokołowski K., Bury W., Justyniak I., Fairen-Jimenez D., Sołtys K., Prochowicz D., Yang S., Schröder M., Lewiński J. (2013). Permanent Porosity Derived from the Self-Assembly of Highly Luminescent Molecular Zinc Carbonate Nanoclusters. Angew. Chem. Int. Ed..

[43] Sokołowski K., Bury W., Justyniak I., Cieślak A.M., Wolska M., Sołtys K., Dzięcielewski I., Lewiński J. (2013). Activation of CO_2_ by TBuZnOH Species: Efficient Routes to Novel Nanomaterials Based on Zinc Carbonates. Chem. Commun..

[44] Leszczyński M.K., Justyniak I., Zelga K., Lewiński J. (2017). From Ethylzinc Guanidinate to [Zn_10_O_4_]-Supertetrahedron. Dalton Trans..

[45] Mąkolski Ł., Szejko V., Zelga K., Tulewicz A., Bernatowicz P., Justyniak I., Lewiński J. (2021). Unravelling Structural Mysteries of Simple Organozinc Alkoxides. Chem. Eur. J..

[46] Lewiński J., Dranka M., Bury W., Śliwiński W., Justyniak I., Lipkowski J. (2007). From Discrete Linear Zn*t*Bu_2_ Molecules to 1D Coordination Polymers and 2D Fabrics. J. Am. Chem. Soc..

[47] Prochowicz D., Sokołowski K., Justyniak I., Kornowicz A., Fairen-Jimenez D., Friščić T., Lewiński J. (2015). A Mechanochemical Strategy for IRMOF Assembly Based on Pre-Designed Oxo-Zinc Precursors. Chem. Commun..

[48] Prochowicz D., Nawrocki J., Terlecki M., Marynowski W., Lewiński J. (2018). Facile Mechanosynthesis of the Archetypal Zn-Based Metal–Organic Frameworks. Inorg. Chem..

[49] Nawrocki J., Prochowicz D., Wiśniewski A., Justyniak I., Goś P., Lewiński J. (2020). Development of an SBU-Based Mechanochemical Approach for Drug-Loaded MOFs. Eur. J. Inorg. Chem..

[50] Lee D., Wolska-Pietkiewicz M., Badoni S., Grala A., Lewiński J., De Paëpe G. (2019). Disclosing Interfaces of ZnO Nanocrystals Using Dynamic Nuclear Polarization: Sol-Gel versus Organometallic Approach. Angew. Chem. Int. Ed..

[51] Krupiński P., Grala A., Wolska-Pietkiewicz M., Danowski W., Justyniak I., Lewiński J. (2021). From Uncommon Ethylzinc Complexes Supported by Ureate Ligands to Water-Soluble ZnO Nanocrystals: A Mechanochemical Approach. ACS Sustain. Chem. Eng..

[52] CrystalExplorer 17.5. crystalexplorer.scb.uwa.edu.au.

[53] Rai V.K., Srivastava R., Chauhan G., Saxena K., Bhardwaj R.K., Chand S., Kamalasanan M.N. (2008). Synthesis and Electroluminescence Properties of Zinc(2,2′-Bipyridine)8-Hydroxyquinoline. Mater. Lett..

[54] Deacon G., Phillips R.J. (1980). Relationships between the Carbon-Oxygen Stretching Frequencies of Carboxylato Complexes and the Type of Carboxylate Coordination. Coord. Chem. Rev..

[55] Li S., Lu J., Ma H., Yan D., Li Z., Qin S., Evans D.G., Duan X. (2012). Luminous Ultrathin Films by the Ordered Micellar Assembly of Neutral Bis(8-Hydroxyquinolate)Zinc with Layered Double Hydroxides. J. Phys. Chem. C.

[56] Brinkmann M., Gadret G., Muccini M., Taliani C., Masciocchi N., Sironi A. (2000). Correlation between Molecular Packing and Optical Properties in Different Crystalline Polymorphs and Amorphous Thin Films of Mer-Tris(8-Hydroxyquinoline)Aluminum(III). J. Am. Chem. Soc..

[57] Otwinowski Z., Minor W. (1997). Processing of X-Ray Diffraction Data Collected in Oscillation Mode. Methods Enzymol..

[58] Agilent Technologies. CrysAlisPro.

[59] Sheldrick G.M. (2008). A Short History of SHELX. Acta Cryst. A.

[60] Spek A.L. (2015). PLATON SQUEEZE: A Tool for the Calculation of the Disordered Solvent Contribution to the Calculated Structure Factors. Acta Cryst. C.

