# Supplementary material for: From a Well-Defined Organozinc Precursor to Diverse Luminescent Coordination Polymers Based on Zn(II)-Quinolinate Building Units Interconnected by Mixed Ligand Systems
Source: Molecules. 2021 Dec 6;26(23):7402. doi: 10.3390/molecules26237402 (PMC8658811; doi:10.3390/molecules26237402)
Supplement: Supplementary file 1 [file molecules-26-07402-s001.zip › molecules-1456958-supplementary.pdf]

molecules

Supporting Information

Article

From a Well-Defined Organozinc Precursor to Diverse Luminescent Coordination Polymers Based on Zn(II)-Quinolinate Building Units Interconnected by Mixed Ligand Systems

Katarzyna Sołtys-Brzostek [1], Kamil Sokołowski [1], Iwona Justyniak [1], Michał K. Leszczyński [1,2], Natalia Olejnik-Fehér [1] and Janusz Lewiński [1,2,*]

1 Institute of Physical Chemistry, Polish Academy of Sciences, Kasprzaka 44/52, 01-224 Warsaw, Poland; ksoltys@ichf.edu.pl (K.S.-B.); ks623@cam.ac.uk (K.S.); ijustyniak@ichf.edu.pl (I.J.); leszczynski.m@hotmail.com (M.K.L.); nolejnik@ichf.edu.pl (N.O.-F.)
2 Faculty of Chemistry, Warsaw University of Technology, Noakowskiego 3, 00-664 Warsaw, Poland
* Correspondence: lewin@ch.pw.edu.pl

X-Ray crystallography

The crystals were selected under Paratone-N oil, mounted on the nylon loops and positioned in the cold stream on the diffractometer. The X-ray data for complex **CP1** were collected on a Nonius Kappa CCD diffractometer using graphite monochromated MoKα radiation (λ = 0.71073 Å). The data were processed with *DENZO* and *SCALEPACK* (*HKL2000* package)[1]. The X-ray data for complexes **CP2**, **CP3**, **HP4**, and **CP5** were collected at 100(2) K on a SuperNova Agilent diffractometer using Mo*Kα* radiation (λ = 0.71073 Å). The data were processed with *CrysAlisPro.*[2] Structures were solved by direct methods and refined using *SHELXL-2016/4.*[3] All non-hydrogen atoms were refined with anisotropic displacement parameters. Hydrogen atoms were added to the structure model at geometrically idealized coordinates and refined as riding atoms.

For compounds CP2, CP3 and HP4 we observed significant residual electron densities within the pores from the solvent molecule. The solvent molecules appeared to be highly disordered and it was difficult to reliably modeled their positions and distribution. Therefore, the *SQUEEZE* function of *PLATON* [4] was used to eliminate the contribution of the electron density in the solvent region from the intensity data.

Due to weak or absent intensity of some reflections, the structure HP4 was fully modeled but has a high wR. Therefore, the geometric parameters were not analyzed.

Crystallographic data (excluding structure factors) have been deposited with the Cambridge Crystallographic Data Centre. Copies of the data can be obtained free of charge on application to CCDC, 12 Union Road, Cambridge CB2 1EZ, UK (fax: +44 1223 336033; e-mail: deposit@ccdc.cam.ac.uk).

CCDC: CP1-2092607, CP2-2092608, CP3-2092609, HP4 -2092610 and CP5 CCDC- 2092611

Table S1. Crystal data and structure refinement details for **CP1**.

Identification code	CP1 CCDC-2092607	
Empirical formula	$C_{42}H_{42}N_4O_9Zn_2$	
Formula weight	877.53	
Temperature	100(2) K	
Wavelength	0.71073 Å	
Crystal system	monoclinic	
Space group	P 21/c	
Unit cell dimensions	a = 10.6080(7) Å	a= 90°.
	b = 9.8830(8) Å	b= 90.954(4)°.
	c = 37.972(3) Å	g = 90°.
Volume	3980.4(5) $Å^3$	
Z	4	
Density (calculated)	1.464 Mg/m^3	
Absorption coefficient	1.266 mm^{-1}	
F(000)	1816	
Crystal size	0.21 x 0.15 x 0.05 mm^3	
Theta range for data collection	2.129 to 22.996°.	
Index ranges	-11<=h<=11, -10<=k<=10, -41<=l<=41	
Reflections collected	7966	
Independent reflections	4801 [R(int) = 0.0693]	
Completeness to theta = 22.996°	86.8 %	
Absorption correction	Semi-empirical from equivalents	
Max. and min. transmission	0.939 and 0.796	
Refinement method	Full-matrix least-squares on F^2	
Data / restraints / parameters	4801 / 29 / 514	
Goodness-of-fit on F^2	1.130	
Final R indices [I>2sigma(I)]	R1 = 0.1072, wR2 = 0.1915	
R indices (all data)	R1 = 0.1663, wR2 = 0.2107	
Extinction coefficient	n/a	
Largest diff. peak and hole	0.986 and -0.454 e.$Å^{-3}$	

Table S2. Crystal data and structure refinement details for **CP2**.

Identification code	CP2 CCDC-2092608	
Empirical formula	$C_{53}H_{53}N_7O_{10}Zn_2$	
Formula weight	1078.76	
Temperature	100(2) K	
Wavelength	0.71073 Å	
Crystal system	Monoclinic	
Space group	P 21/n	
Unit cell dimensions	a = 10.260(5) Å	a= 90.000(5)°
	b = 21.650(5) Å	b= 91.910(5)°.
	c = 25.883(5) Å	g = 90.000(5)°.
Volume	5746(3) Å ^{3}	
Z	4	
Density (calculated)	1.247 Mg/m^3	
Absorption coefficient	0.893 mm^{-1}	
F(000)	2240	
Crystal size	0.17 x 0.12 x 0.06 mm^3	
Theta range for data collection	1.226 to 25.677°.	
Index ranges	-12<=h<=11, -26<=k<=25, -27<=l<=31	
Reflections collected	10862	
Independent reflections	10862 [R(int) = 0.108]	
Completeness to theta = 25.677°	99.9 %	
Absorption correction	Semi-empirical from equivalents	
Max. and min. transmission	0.939 and 0.796	
Refinement method	Full-matrix least-squares on F^2	
Data / restraints / parameters	10862 / 0 / 702	
Goodness-of-fit on F^2	1.099	
Final R indices [I>2sigma(I)]	R1 = 0.0733, wR2 = 0.2136	
R indices (all data)	R1 = 0.0866, wR2 = 0.2265	
Largest diff. peak and hole	0.862 and -0.882 e.Å $^{-3}$	

Table S3. Crystal data and structure refinement details for **CP3**.

Identification code	CP3 CCDC-2092609	
Empirical formula	$C_{47}H_{37}N_5O_8Zn_2$	
Formula weight	930.55	
Temperature	100(2) K	
Wavelength	0.71073 Å	
Crystal system	triclinic	
Space group	P -1	
Unit cell dimensions	a = 13.969(2) Å	a= 85.971(12)°
	b = 14.456(3) Å	b= 63.139(13)°.
	c = 14.6261(14) Å	g = 73.145(17)°.
Volume	2515.2(8) Å ^{3}	
Z	2	
Density (calculated)	1.229 Mg/m^3	
Absorption coefficient	1.005 mm^{-1}	
F(000)	956	
Crystal size	0.19 x 0.10 x 0.08 mm^3	
Theta range for data collection	3.043 to 25.682°.	
Index ranges	-16<=h<=17, -17<=k<=15, -17<=l<=17	
Reflections collected	14812	
Independent reflections	8248 [R(int) = 0.1116]	
Completeness to theta = 25.682°	86.93 %	
Absorption correction	Semi-empirical from equivalents	
Max. and min. transmission	0.886 and 0.923	
Refinement method	Full-matrix least-squares on F^2	
Data / restraints / parameters	8248 / 0 / 561	
Goodness-of-fit on F^2	0.931	
Final R indices [I>2sigma(I)]	R1 = 0.1044, wR2 = 0.2145	
R indices (all data)	R1 = 0.2262, wR2 = 0.2723	
Largest diff. peak and hole	1.101 and -0.540 e.Å $^{-3}$	

Table S4. Crystal data and structure refinement details for **HP4**.

Identification code	HP4 CCDC- 2092610	
Empirical formula	$C_{114}H_{98}N_{14}O_{20}Zn_4$	
Formula weight	2245.54	
Temperature	100(2) K	
Wavelength	0.71073 Å	
Crystal system	monoclinic	
Space group	C 2/c	
Unit cell dimensions	a = 18.5180(15) Å	a= 90°
	b = 18.500(3) Å	b= 94.015(6)°.
	c = 34.4199(17) Å	g = 90°.
Volume	11763(2) Å ^{3}	
Z	4	
Density (calculated)	1.268 Mg/m^3	
Absorption coefficient	0.875 mm^{-1}	
F(000)	4640	
Crystal size	0.18 x 0.09 x 0.06 mm^3	
Theta range for data collection	3.144 to 24.499°.	
Index ranges	-24<=h<=23, -14<=k<=24, -25<=l<=46	
Reflections collected	9748	
Independent reflections	9748 [R(int) = 0.0912]	
Completeness to theta = 24.499°	99.6 %	
Absorption correction	Semi-empirical from equivalents	
Max. and min. transmission	0.910 and 0.949	
Refinement method	Full-matrix least-squares on F^2	
Data / restraints / parameters	9748/ 39 / 708	
Goodness-of-fit on F^2	1.237	
Final R indices [I>2sigma(I)]	R1 = 0.1477, wR2 = 0.3767	
R indices (all data)	R1 = 0.2041, wR2 = 0.4077	
Largest diff. peak and hole	2.818 and - -0.806 e.Å $^{-3}$	

Table S4. Crystal data and structure refinement details for **CP5**.

Identification code	CP5 CCDC- 2092611	
Empirical formula	$C_{17}H_{17}N_4O_5Zn$	
Formula weight	422.71	
Temperature	100(2) K	
Wavelength	0.71073 Å	
Crystal system	triclinic	
Space group	P -1	
Unit cell dimensions	a = 9.921(3) Å	a= 87.37(3)°.
	b = 10.137(4) Å	b= 73.13(3)°.
	c = 11.012(4) Å	g = 60.77(4)°.
Volume	918.8(7) Å ^{3}	
Z	2	
Density (calculated)	1.528 Mg/m^3	
Absorption coefficient	1.373 mm^{-1}	
F(000)	434	
Crystal size	0.18 x 0.14 x 0.10 mm^3	
Theta range for data collection	3.193 to 29.064°.	
Index ranges	-12<=h<=13, -12<=k<=13, -14<=l<=15	
Reflections collected	6754	
Independent reflections	4158 [R(int) = 0.0387]	
Completeness to theta = 29.064°	85.0 %	
Absorption correction	Semi-empirical from equivalents	
Max. and min. transmission	0.794 and 0.872	
Refinement method	Full-matrix least-squares on F^2	
Data / restraints / parameters	4158 / 175 / 291	
Goodness-of-fit on F^2	1.030	
Final R indices [I>2sigma(I)]	R1 = 0.0481, wR2 = 0.1124	
R indices (all data)	R1 = 0.0575, wR2 = 0.1186	
Largest diff. peak and hole	1.141 and -1.170 e.Å $^{-3}$	

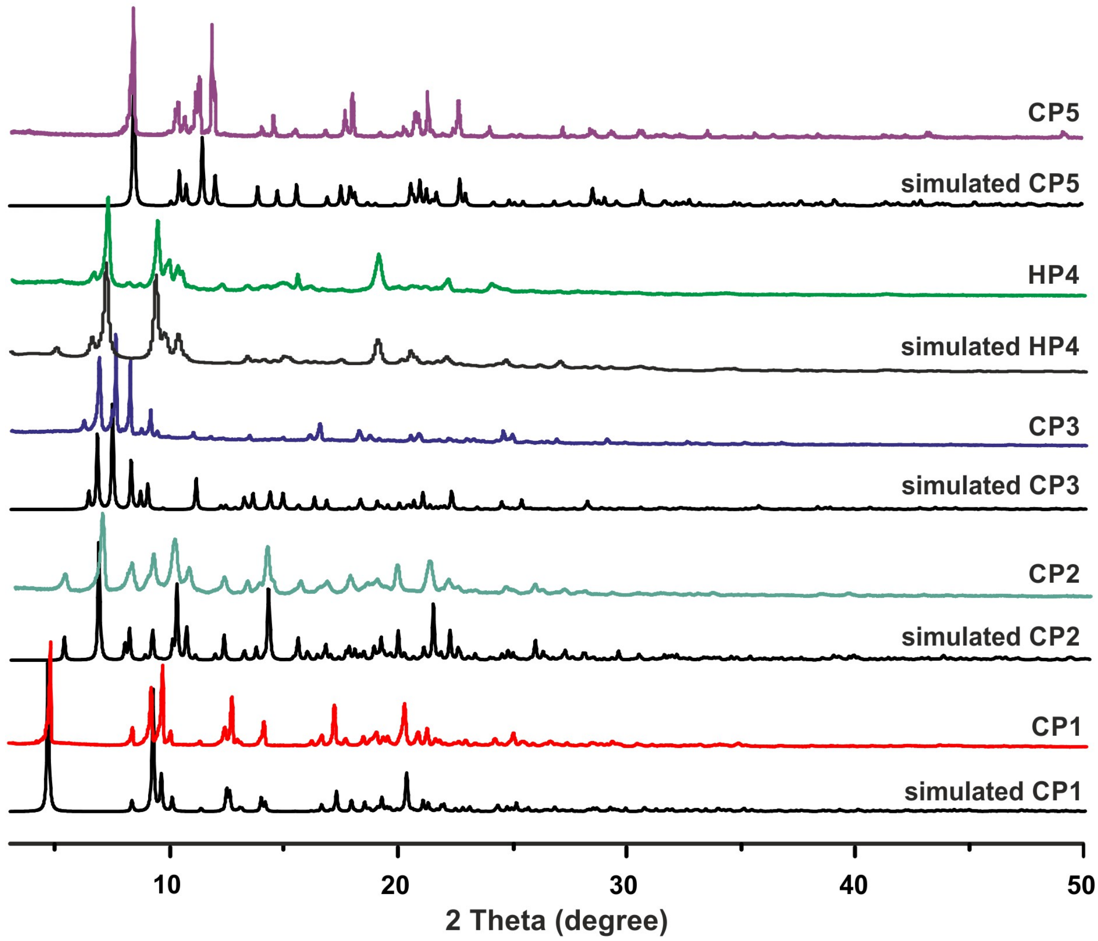

Figure S1. Experimental and simulated PXRD patterns for the **CP1**-**CP5** and **HP4** polymers. Simulation of PXRD pattern for **HP4** involved refinement of preferred orientation due to significant texturing in the sample.

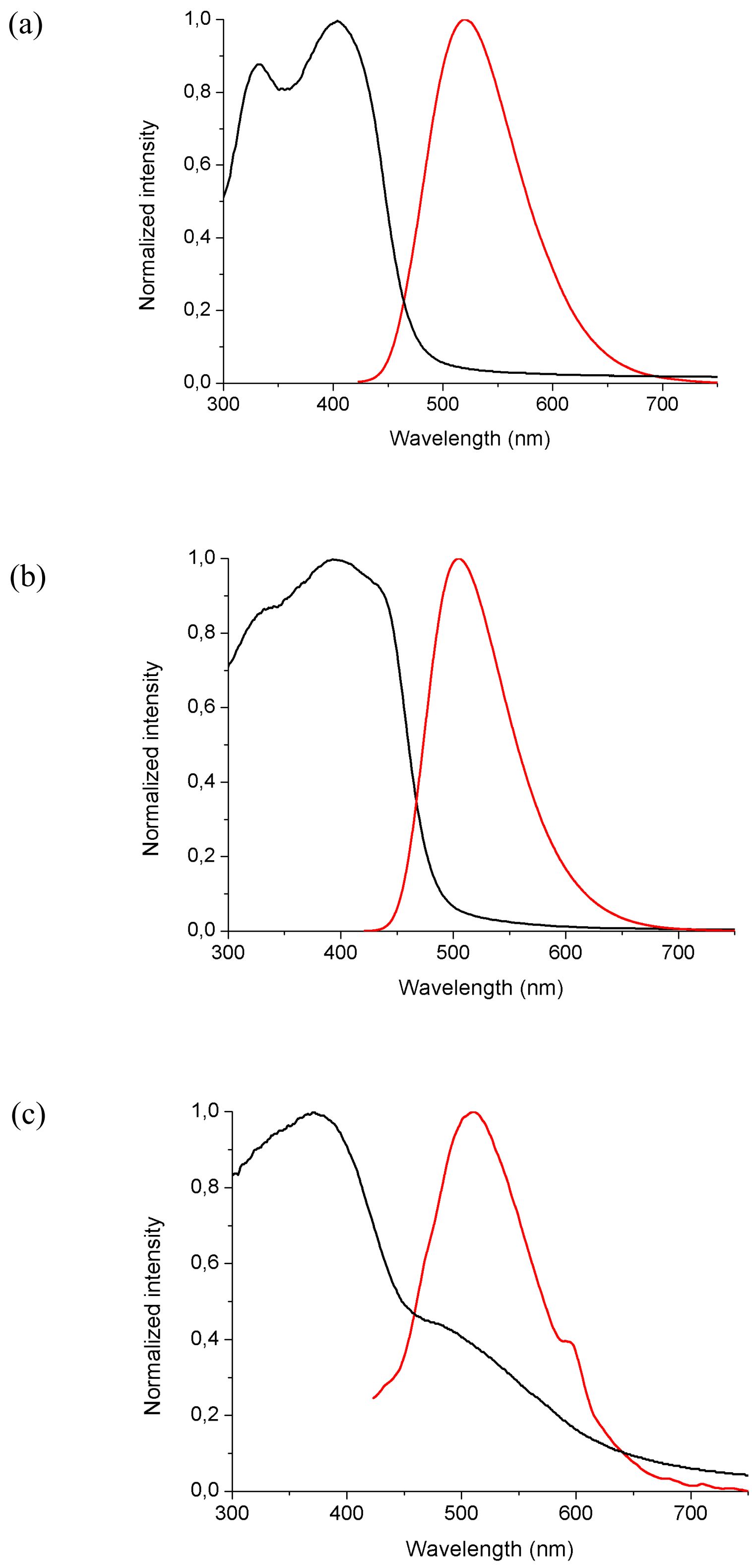
(a)
1,0
0,8
0,6
0,4
0,2
0,0
Normalized intensity
300
400
500
600
700
Wavelength (nm)
(b)
1,0
0,8
0,6
0,4
0,2
0,0
Normalized intensity
300
400
500
600
700
Wavelength (nm)
(c)
1,0
0,8
0,6
0,4
0,2
0,0
Normalized intensity
300
400
500
600
700
Wavelength (nm)

(d)

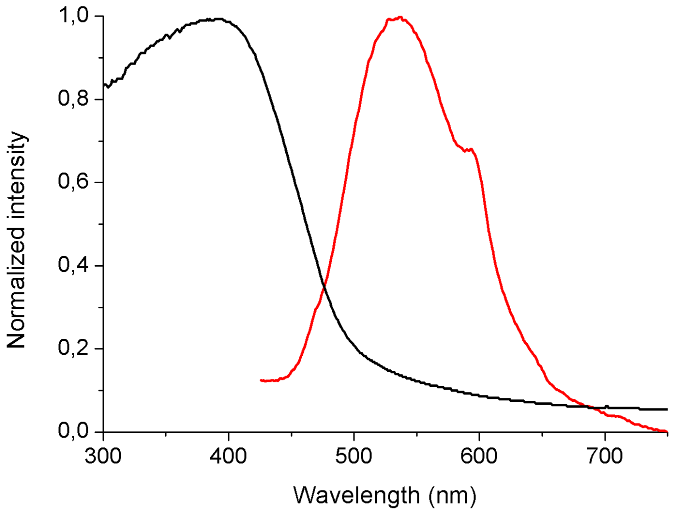

Figure S2. UV-Vis absorption measured in diffusive reflectance of polymer powders dispersed with $BaSO_4$ (black line) and the photoluminescence solid-state spectra of polymers measured with 400 nm excitation (red line): (a) **CP1**, (b) **CP2**, (c) **CP3**, (d) **HP4**.

References

(1) Z. Otwinowski, W. Minor, *Methods Enzymol*. **1997**, *276*, 307-326.
(2) Agilent Technologies, CrysAlisPro, Version 1.171.35.21b
(3) Sheldrick, G. M. A short history of SHELX. *Acta Crystallogr.* **2008**, *64A*, 112-122.
(4) Spek, A. L. *Acta Crystallogr.* **2015**, *C71*, 9-18.
